# Sputter-prepared (001) BiFeO_3_ thin films with ferromagnetic L1_0_-FePt(001) electrode on glass substrates

**DOI:** 10.1186/1556-276X-7-435

**Published:** 2012-08-03

**Authors:** Huang-Wei Chang, Fu-Te Yuan, Chih-Wei Shih, Ching-Shun Ku, Ping-Han Chen, Chang-Ren Wang, Wen-Cheng Chang, Shien-Uang Jen, Hsin-Yi Lee

**Affiliations:** 1 Department of Physics, Tunghai University, Taichung, 407, Taiwan; 2 Department of Physics, National Taiwan University, Taipei, 106, Taiwan; 3 Department of Physics, National Chung Cheng University, Chia-Yi, 621, Taiwan; 4 National Synchrotron Radiation Research Center, Hsinchu, 300, Taiwan; 5 Institute of Physics, Academia Sinica, Taipei, 115, Taiwan

**Keywords:** Multiferroic BiFeO3 (001) films, L10-FePt(001) underlayer, Glass substrate

## Abstract

Highly textured BiFeO_3_(001) films were formed on L1_0_-FePt(001) bottom electrodes on glass substrates by sputtering at reduced temperature of 400°C. Good electric polarization 2*P*_r_ = 80 and 95 μC/cm^2^, comparable to that of the reported epitaxial films, and coercivity *E*_c_ = 415 and 435 kV/cm are achieved in the samples with 20-nm- and 30-nm-thick electrodes. The BiFeO_3_(001) films show different degrees of compressive strain. The relation between the variations of strain and 2*P*_r_ suggests that the enhancement of 2*P*_r_ resulted from the strain-induced rotation of spontaneous polarization. The presented results open possibilities for the applications based on electric-magnetic interactions.

## Background

BiFeO_3_ (BFO) with a rhombohedral perovskite structure has attracted considerable attention due to its multiferroic properties above room temperature (RT) including high ferroelectric (*T*_C_ = 830°C) and G-type antiferromagnetic (AFM) (*T*_N_ = 370°C) transition temperatures [1-4]. Different from the spiral spin structure in bulk, BFO thin film exhibits an antiparallel AFM structure along [111], allowing coupling to the spins of a ferromagnetic (FM) layer at the interface. The coupling permits the possibilities of various advanced spintronic and memory devices based on the electric-magnetic interactions [2-5].

Ferroelectric properties of BFO films highly depend on preferred orientation [6-11]. (111)-textured BFO shows the highest remanent polarization 2*P*_r_ of approximately 200 μC/cm^2^[6-10]. Nevertheless, BFO(001) (2*P*_r_ = 40 to 120 μC/cm^2^) shows more advantages for practical uses, such as lower electrical coercive field (*E*_c_), better fatigue resistance, and higher piezoelectric coefficient [9-12]. For the BFO films prepared by either pulsed laser deposition (PLD) or sputtering, the preferred orientation can be well controlled by either using proper single crystal substrates or controlling the texture of the perovskite electrode underlayers [2,5-13].

However, the high processing temperature (*T*_p_ > 600°C) [6-10] as well as the cost of using perovskite substrates is not favorable to industry. Although it has been reported that the use of the metal electrode Pt can reduce *T*_p_ to about 500°C, single crystal substrates are still necessary for texture control of both Pt and BFO [11]. Considering that the electric-magnetic coupling is the fundamental mechanism to function the related spintronic devices, development of FM electrode that can induce a specific texture of BFO is thus one of the most effective ways to facilitate this coupling.

However, no related investigation has been reported prior to the presented study. In this letter, we demonstrate the induction of the BFO(001) preferred orientation for the sputter-prepared thin films by strongly textured ferromagnetic electrode of L1_0_(001) FePt on glass substrates. Structural as well as ferroelectric properties are reported in detail.

## Methods

**Figure 1:**
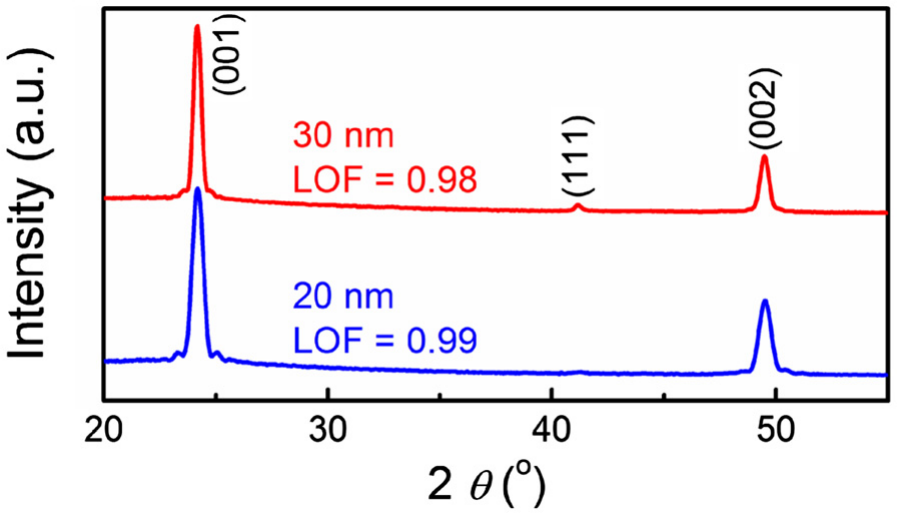
**XRD patterns of the optimized 20- and 30-nm-thick L1**_**0**_-**FePt bottom electrodes.**

The selection of L1_0_-FePt(001) as a bottom electrode is due to the similar lattice parameter between L1_0_-FePt (*a* = 3.86 Å) [14] and pseudocubic BFO (*a* = 3.965 Å) [3]. Appropriate lattice mismatch is expectedly advantageous for the induction of BFO(001). The bilayer films of BFO(001)/FePt(001) were prepared by sputtering with a base pressure better than 5 × 10^−7^ Torr. The FePt electrode underlayer with thicknesses of 20 and 30 nm was firstly deposited on Corning 1737 glass substrates (Corning Inc., Corning, NY, USA) at RT and then submitted to rapid thermal annealing at temperatures ranging from 500°C to 800°C, a heating rate of 40°C/s for 0 to 20 min, and a pressure of 2 × 10^−6^ Torr to form highly ordered L1_0_ phase with strong (001) texture via grain growth dominated by strong densification tensile stress [15]. Further increase in the FePt film thickness leads to isotropic growth of the L1_0_ grains [16]. X-ray diffraction (XRD) patterns of the optimized 20- and 30-nm-thick L1_0_-FePt films are shown in Figure 1. Strong (001) and (002) peaks with fringes indicate good texture and smooth surface of the electrode. The degree of texture is quantified by Lotgering orientation factor (LOF), an index of a specific preferred orientation like {00 *l*} expressed as LOF = (*p* − *p*_0_)/(1 − *p*_0_), where *p* = Σ(00 *l*)_film_/Σ(*hkl*)_film_ and *p*_0_ = Σ(00 *l*)_powder_/Σ(*hkl*)_powder_[17]. LOF varies from 0 for a randomly oriented sample to 1 for a completely oriented sample. Values of 0.99 and 0.98 were obtained for the optimized FePt electrodes with 20 and 30 nm in thickness, respectively. Additionally, root-mean-square surface roughness (*R*_rms_) of the electrode layers measured by atomic force microscopy (AFM) is less than 1 nm. After the preparation of the FePt(001) bottom electrode, the BFO layer was deposited at a low substrate temperature (*T*_d_) of 400°C using a commercial Bi_1.1_FeO_3_ target. Due to the low deposition temperature of BFO, a compositional sharp interface between the FePt(001) bottom electrode and BFO layer is expected, which is important to both the development of BFO(001) texture and the FM/AFM interactions. The working pressure is set as 10 mTorr, and the ratio of Ar to O_2_ is 4 to 1. The structure of the films was characterized by high-resolution X-ray diffraction (HRXRD) and normal XRD technique. HRXRD and residual strain measurements were conducted at synchrotron wiggler beamline BL-17B1 in the Taiwan Light Source of the National Synchrotron Radiation Research Center, Hsinchu, Taiwan. The use of two pairs of slits between the sample and the detector provided a typical wave vector resolution of approximately 0.001 nm^−1^ in the vertical scattering plane. Surface morphology was observed by scanning electron microscopy (SEM) and AFM. For electric property measurement, circular Au top electrodes of 500 μm in diameter were sputtered onto the film surface using a shadow mask. The ferroelectric properties at RT were measured using the TF 2000 Analyzer FE-Module (axiACCT Systems GmbH, Aachen, Germany) ferroelectric test system at frequencies of 1 kHz.

## Results and discussion

**Figure 2:**
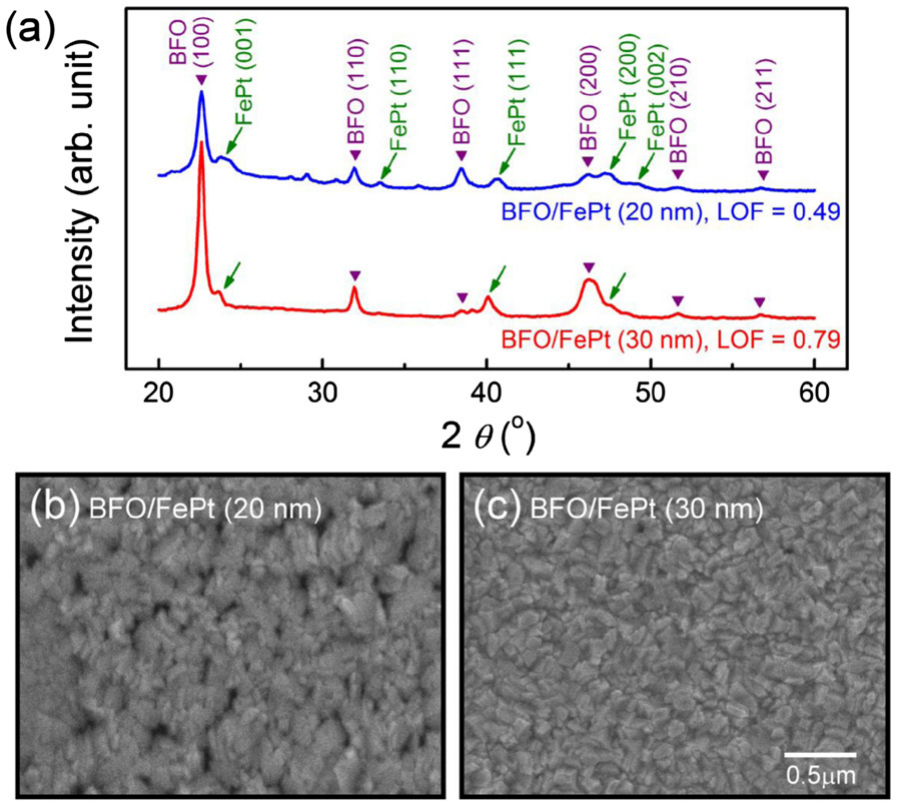
**HRXRD patterns and SEM images.** (**a**) HRXRD patterns of 200-nm-thick BFO films grown on 20- and 30-nm-thick L1_0_-FePt bottom electrodes at a *T*_d_ of 400 °C. SEM images of BFO films with (**b**) 20- and (**c**) 30-nm-thick L1_0_-FePt bottom electrodes.

Figure 2a depicts HRXRD patterns of BFO/FePt films with electrode thicknesses (*t*_e_) of 20 and 30 nm grown at *T*_d_ = 400°C. The single phase of the pseudocubic perovskite was confirmed by the presented BFO peaks in both samples. In the sample with 20-nm-thick FePt electrode, the intensity of diffractions other than (00 *l*) is stronger than those of the 30-nm-thick FePt underlayered film. The LOF values of the BFO films with 20- and 30-nm-thick FePt bottom electrodes determined by the integrated intensity of the peaks in the range of 2*θ* from 20° to 60° are 0.49 and 0.79, respectively; the larger value is similar to the published data for the BFO epitaxial film grown on SrTiO_3_(001) surface by PLD (LOF approximately 0.75) [18]. The lower LOF of the sample with thinner electrode is believed to result from the degraded (001) texture of FePt as evidenced by the presence of the L1_0_(110), L1_0_(111), and L1_0_(200) peaks, which are not shown before the deposition of the BFO layer. The degeneration of the L1_0_(001) preferred orientation, possibly a result of residual stress/strain relaxation, is not obvious in the specimen with thicker electrode. Figure 2b,c shows SEM images for the 200-nm-thick BFO films grown on 20- and 30-nm-thick L1_0_-FePt electrodes, respectively. Densely packed grains with average size in the range of 50 to 150 nm is observed in both samples, and no crack is found. The surface roughness of the films is in the range of 4 to 6 nm, but the sample with thicker electrode shows more uniform surface morphology. The above results indicate that although the FePt electrodes with different thicknesses exhibit similar texture before the growth of BFO layer, only the 30-nm-thick electrode achieves good BFO(001) texture.

**Figure 3:**
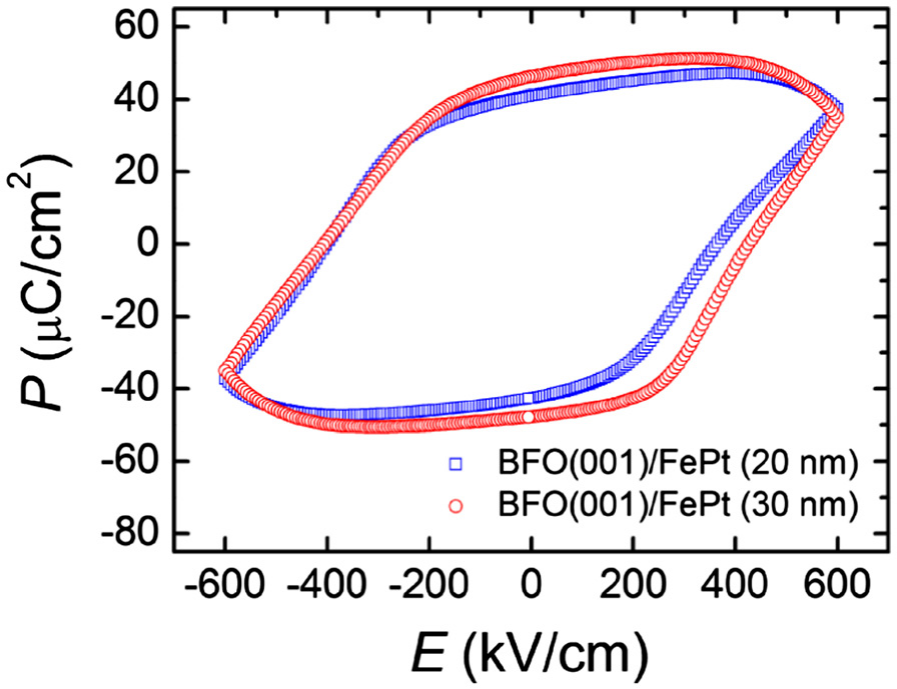
**Electrical P-E curves of 200-nm-thick BFO films grown on 20- and 30-nm-thick L1**_**0**_-**FePt bottom electrodes.** The films were grown at a *T*_d_ of 400°C.

Ferroelectric properties of 200-nm-thick BFO films with bottom electrodes of 20 nm and 30 nm in thickness are shown in Figure 3. Values of 2*P*_r_ = 80 μC/cm^2^ and *E*_c_ = 385 kV/cm for the 20-nm-thick FePt underlayered BFO film and 2*P*_r_ = 95 μC/cm^2^ and *E*_c_ = 415 kV/cm for the one with 30-nm-thick electrode are obtained. The *P*_r_ values are comparable to those of epitaxial BFO(001) films grown on a SrRuO_3_/SrTiO_3_(001) and Pt/MgO(100) substrates; however, the *E*_c_ values are significantly higher than values of those films (*E*_c_ approximately 200 kV/cm) [2,6-13]. In addition to the large *E*_c_, the hysteresis loops are rounded as compared to the rectangular-shaped loops of the films using single crystal substrates. The different hysteresis behaviors and properties from the epitaxial film may be related to the reversal process of electric polarization. For the films grown on a specific plane of a single crystal, the movement of the ferroelectric domain wall tends to be continuous due to the alignment of both in-plane and out-of-plane orientation of lattice, resulting in sharp switching of polarization. In contrast, the presented L1_0_ electrode aligns only the out-of-plane (001) orientation of BFO; the random distribution of the in-plane orientation as well as the small grain size comparing to the diameter of the top electrode (500 μm) expectedly reduce the continuity of the domain wall motion, leading to increased *E*_c_ and rounded hysteresis loop. The effect of coercivity enhancement with rounded loops has also been reported in sputtered BFO films using metal bottom electrode [19]. In order to investigate the magnetic interactions between the FM electrode and AFM BFO layer, polarization-electric field (P-E) hysteresis loops were measured with the application of a magnetic field. It is observed that the polarization of FePt(001)/BFO measured under an external magnetic field of 3.5 kOe is enhanced by 9% as compared to that obtained at zero magnetic field. This result provides unambiguous evidence for the strong FM/AFM coupling between FePt and BFO layer. Detailed measurements are still undergoing, and the mechanism remains to be clarified.

**Figure 4:**
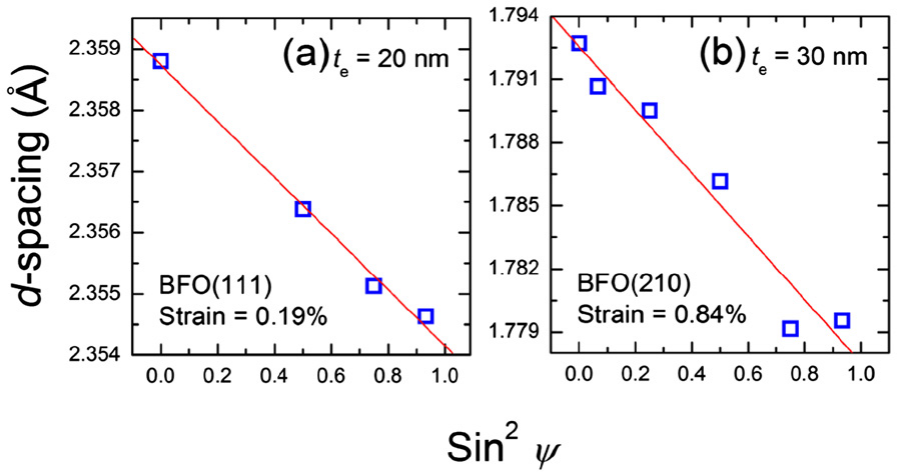
**Dependence of residual strain on sin**^**2**^***ψ *for 200-nm-thick BFO films.** The films were grown on (**a**) 20- and (**b**) 30-nm-thick L1_0_-FePt bottom electrodes at a *T*_d_ of 400 °C.

Although controlling the texture of BFO films using a metal underlayer shows advantage of lowering formation temperature as reported in the BFO(001)/Pt/MgO(100) system [11], the value of *P*_r_ is reduced. Similar *P*_r_ reduction can also been observed in the BFO films with random orientation grown on the isotropic Pt underlayer [10,20,21]. To further understand the reason for the relatively higher *P*_r_ in the presented study, we investigate the residual biaxial strain of the BFO(001) films because it has been observed in a number of compounds that strong coupling between strain and ferroelectric properties in ferroelectrics results in significant enhancement in polarization and Curie temperature. For BFO(111) films, theoretical studies of both thermodynamics and first-principle predicted a negligible effect of strain on polarization [22,23]; however, the experimental results confirm that in BFO(001), the biaxial strain induces a rotation of spontaneous polarization, resulting in drastic increase in *P*_r_[13]. The expected increment is as high as 25% when the compressive strain reaches 1%. The presented residual stain of BFO(001) was measured by Sin^2^*ψ* method [24]. (111) and (210) peaks are selected for the BFO films grown on 20-nm- and 30-nm-thick electrodes, respectively, as indexed to extract biaxial strain for signal optimization. The dependences of planar spacing on Sin^2^*ψ* are shown in Figure 4. Good linearity are obtained in both samples, indicative of uniform strain state along plane normal, that is, negligible strain relaxation across the film. Compressive residual strain is confirmed in both BFO films, which is considered responsible for the presented *P*_r_ values. Large compressive strain of 0.84%, higher than that induced by the SrTiO_3_(001) underlayer/substrate (approximately 0.55%) [13], obtained in the film with 30-nm-thick electrode is attributed to the smaller lattice parameter *a* ≈ 3.86 Å of L1_0_-FePt compared to that of SrTiO_3_(001) *a* ≈ 3.9 Å, producing a larger lattice mismatch of −2.6%. However, the strain decreases to 0.19% in the film with 20-nm-thick electrode. The strain relaxation of the BFO layer is believed to result from the simultaneous changes in the electrode layer during the deposition of BFO at 400°C as described earlier. With the increase of compressive strain from 0.19% to 0.84%, *P*_r_ is enhanced by 18.7%, which is close to the increment of 15.5% deduced from the linear dependence of *P*_r_ on the in-plane strain predicted by theoretical thermodynamic analysis [13]. The result corroborates the validity of *P*_r_ enhancement which resulted from the previously proposed mechanism of spontaneous polarization rotation induced by the strain [13] in the presented BFO(001)/FePt(001) system.

**Figure 5:**
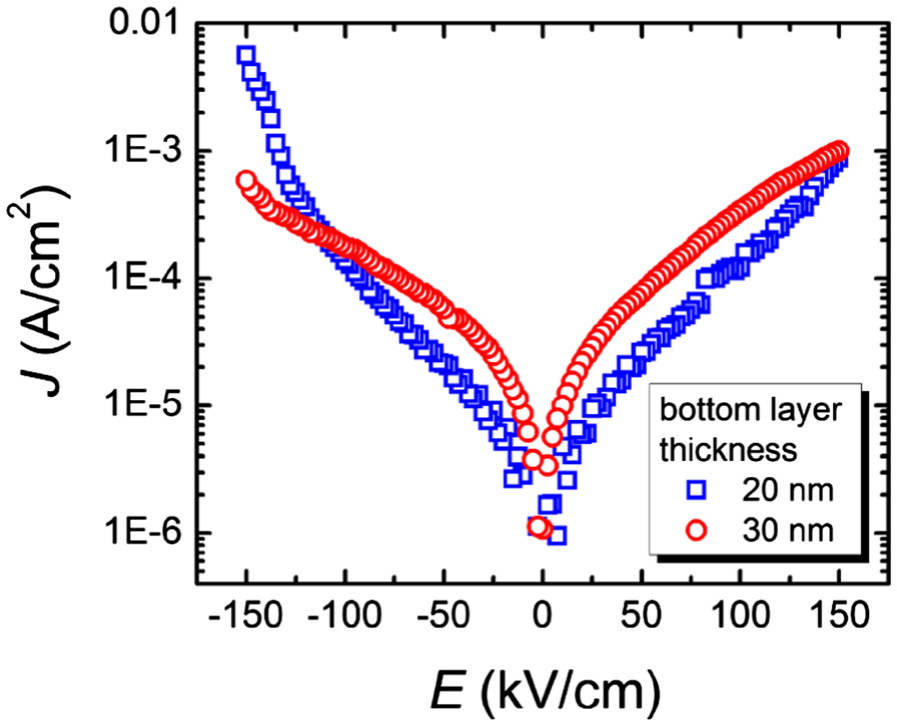
**Dependence of current density on applied electric field of 200-nm-thick BFO films.** The films were grown on 20- and 30-nm-thick L1_0_-FePt bottom electrodes at a *T*_d_ of 400°C.

Current density *J* as a function of external electric field is shown in Figure 5. Although the sample with 30-nm-thick FePt underlayer has enhanced polarization, leakage current is high. A relatively smaller leakage current was obtained in the sample with 20-nm-thick FePt electrode. Comparing to the results of internal strain, a relation that the leakage current is inversely proportional to the compressive strain can be established. This relation agrees well with the results obtained in epitaxial BFO films [9]. The explanation for this needs further confirmation. It is worthy noting that the present value of *J* is more than two orders of magnitude smaller than that of the reported sample prepared by sputtering using SrRuO_3_/Pt buffer layers [10]. The reported results manifest that FePt(001) is a highly potential electrode for both future application and scientific research.

## Conclusions

The induction of strong (001) texture of BFO films using the ferromagnetic FePt(001) bottom electrode with thicknesses of 20 and 30 nm on glass substrates by rf sputtering is reported. A degree of preferred orientation (LOF = 0.79) higher than that of the film prepared by PLD on SrTiO_3_(001) is achieved in the sample with 30-nm-thick electrode at a reduced temperature of 400°C. 2*P*_r_ values of 80 and 95 μC/cm^2^ are obtained in the films with 20-nm- and 30-nm-thck electrodes, respectively, much higher than that of the BFO(001) epitaxial films with Pt(001) bottom electrode grown on single crystal substrates [11]. The BFO(001) films with 20-nm- and 30-nm-thick electrodes exhibit different compressive strains of 0.19% and 0.84%, respectively, and the relation between the increments of 2*P*_r_ and biaxial strain suggests that the strain-induced polarization rotation mechanism reported previously [13] is responsible for the variation of 2*P*_r_. The results of this study demonstrate the advantages of fabricating BFO(001) films using ferromagnetic bottom electrode on non-textured substrates and open wide possibilities for advanced applications based on electric-magnetic couplings.

## Competing interests

The authors declare that they have no competing interests.

## References

[B1] Teague JR Gerson R James WJ Dielectric hysteresis in single crystal BiFeO3 Solid State Commun 1970 7 1073 10.1016/0038-1098(70)90262-0

[B2] Wang J Neaton JB Zheng H Nagarajan V Ogale SB Liu B Viehland D Vaithyanathan V Schlom DG Waghmare UV Spaldin NA Rabe KM Wuttig M Ramesh R Epitaxial BiFeO3 multiferroic thin film heterostructures Science 2003 7 1719 10.1126/science.1080615 12637741

[B3] Catalan G Scott JF Physics and applications of bismuth ferrite Adv Mater 2009 7 2463 10.1002/adma.200802849

[B4] Bea H Gajek M Bibe M Barthelemy A Spintronics with multiferroics J Phys Condens Matter 2008 7 434221 10.1088/0953-8984/20/43/434221

[B5] Bea H Bibes M Fusil S Bouzehouane K Jacquet E Rode K Bencok P Barthelemy A Investigation on the origin of the magnetic moment of BiFeO3 thin films by advanced x- ray characterizations Phys. Rev. B 2006 7 020101

[B6] Li J Wang J Wuttig M Ramesh R Wang N Ruette B Pyatakov AP Zvezdin AK Viehland D Dramatically enhanced polarization in (001), (101), and (111) BiFeO3 thin films due to epitiaxial-induced transitions Appl Phys Lett 2004 7 5261 10.1063/1.1764944

[B7] Das RR Kim DM Beak SH Eom CB Zavaliche F Yang SY Ramesh R Chen YB Pan XQ Ke X Rzchowski MS Streiffer SK Synthesis and ferroelectric properties of epitaxial BiFeO3 thin films grown by sputtering Appl Phys Lett 2006 7 242904 10.1063/1.2213347

[B8] Chu YH Martin LW Holcomb MB Ramesh R Controlling magnetism with multiferroics Mater. Today 2007 7 16

[B9] Jang HW Beak SH Ortiz D Folkman CM Eom CB Chu YH Shafer P Ramesh R Vaithyanathan V Schlom DG Epitaxial (001) BiFeO3 membranes with substantially reduced fatigue and leakage Appl Phys Lett 2008 7 062910 10.1063/1.2842418

[B10] Wu J Wang J Orientation dependence of ferroelectric behavior of BiFeO3 thin films J Appl Phys 2009 7 104111 10.1063/1.3261841

[B11] Ryu S Son JY Shih YH Jang HM Scott JF Polarization switching characteristics of BiFeO3 thin films epitaxially grown on Pt/MgO at a low temperature Appl Phys Lett 2009 7 242902 10.1063/1.3275012

[B12] Wang J Zheng H Ma Z Prasertchoung S Wuttig M Droopad R Yu J Eisenbeiser K Ramesh R Epitaxial BiFeO3 thin films on Si Appl Phys Lett 2004 7 2574 10.1063/1.1799234

[B13] Jang HW Beak SH Ortiz D Folkman CM Das RR Chu YH Shafer P Zhang JX Choudhury S Vaithyanathan V Chen YB Felker DA Biegalski MD Rzchowski MS Pan XQ Schlom DG Chen LQ Ramesh R Eom CB Strain-induced polarization rotation in epitaxial (001) BiFeO3 thin films Phys Rev Lett 2008 7 107602 18851256 10.1103/PhysRevLett.101.107602

[B14] Villars P Calvert LD Pearson's Handbook of Crystallographic Data for Intermetallic Phase 2000 Meta Park, OH: ASM

[B15] Hsiao N Yuan FT Chang HW Huang HW Chen SK Lee HY Effect of initial stress/strain state on order–disorder transformation of FePt thin films Appl Phys Lett 2009 7 232505 10.1063/1.3153513

[B16] Mei JK Yuan FT Liao WM Sun AC Yao YD Lin HM Hsu JH Lee HY Critical thickness of (001) texture induction in FePt thin films on glass substrates IEEE Trans Magn 2011 7 3633

[B17] Lotgering FK Topotactical reactions with ferrimagnetic oxides having hexagonal crystal structures-I J Inorg Nucl Chem 1959 7 113 10.1016/0022-1902(59)80070-1

[B18] Bark CW Cho KC Koo YM Tamura N Ryu S Jang HM Two-dimensional mapping of triaxial strain fields in a multiferroic BiFeO3 thin film using scanning x-ray microdiffraction Appl Phys Lett 2007 7 102904 10.1063/1.2711530

[B19] Zheng RY Gao XS Zhou ZH Wang J Multiferroic BiFeO3 thin films deposited on SrRuO3 buffer layer by rf sputtering J Appl Phys 2007 7 054104 10.1063/1.2437163

[B20] Lee YH Wu JM Chueh YL Chou LJ Low-temperature growth and interface characterization of BiFeO3 thin films with reduced leakage current Appl Phys Lett 2005 7 172901 10.1063/1.2112181

[B21] Lee YH Wu JM Lai CH Influence of La doping in multiferroic properties of BiFeO3 thin films Appl Phys Lett 2006 7 042903 10.1063/1.2167793

[B22] Ederer C Spaldin NA Effect of epitaxial strain on the spontaneous polarization of thin film ferroelectrics Phys Rev Lett 2005 7 257601 16384507 10.1103/PhysRevLett.95.257601

[B23] Zhang JX Li YL Wang Y Liu ZK Chen LQ Chu YH Zavaliche F Ramesh R Effect of substrate-induced strains on the spontaneous polarization of epitaxial BiFeO3 thin films J Appl Phys 2007 7 114105 10.1063/1.2743733

[B24] Fitzpatric ME, Lodini A Analysis Of Residual Stress By Diffraction Using Neutron And Synchrotron Radiation 2003 London: Taylor & Francis

